# Men at risk of gonococcal urethritis: a case-control study in a Darwin sexual health clinic

**DOI:** 10.1186/s12879-019-4625-8

**Published:** 2019-11-21

**Authors:** Winnie Chen, Suzanne Connor, Manoji Gunathilake

**Affiliations:** 1 Sexual Health and Blood Borne Virus Unit, Centre for Disease Control (NT), Ground Floor Building 4 Royal Darwin Hospital Rocklands Drive, Tiwi, NT 0810 Australia; 2 Kirby Institute, Level 6, Wallace Wurth Building High Street, UNSW, Kensington, NSW 2052 Australia

**Keywords:** Urethritis, Men, *Neisseria gonorrhoeae*, *Chlamydia trachomatis*, Ceftriaxone, Antimicrobial resistance

## Abstract

**Background:**

Male urethritis is primary sexually transmitted. Northern Territory (NT) has the highest rates of gonococcal infection in Australia and local guidelines recommend empiric treatment with azithromycin and ceftriaxone for all men presenting with urethritis. As gonococcal drug resistance is a growing concern, this study aims to improve empiric use of ceftriaxone through examining local patterns of male urethritis, comparing cases of gonococcal urethritis (GU) to controls with non-gonococcal urethritis (NGU).

**Methods:**

A retrospective study was undertaken of all men with symptomatic urethritis presenting to Darwin sexual health clinic from July 2015 to July 2016 and aetiology of urethritis in this population was described. Demographic, risk profile, and clinical features of GU cases were compared to NGU controls.

**Results:**

Among *n* = 145 men, the most common organisms identified were *Chlamydia trachomatis* (23.4%, SE 3.5%) and *Neisseria gonorrhoeae* (17.2%, SE 3.1%). The main predictors of GU were any abnormalities on genital examination (aOR 10.4, 95% CI 2.1 to 50.8) and a history of urethral discharge (aOR 5.7, 95% CI 1.4 to 22.6). Aboriginal patients (aOR 3.0, 95% CI 0.9 to 9.6) and those over 30 years of age (aOR 1.4, 95% CI 0.3 to 7.0) were more likely to have GU in the unadjusted analysis, but not in the adjusted model.

**Conclusion:**

This is the first study looking at patterns of male urethritis in urban NT and the results support a move towards adopting national guidelines to use ceftriaxone for empiric management of syndromic urethritis only in high-risk patients. In addition to traditional demographic risk factors, clinical features remain an important component of risk stratification.

## Introduction

Sexually transmitted infections account for the majority of male urethritis and symptoms include urethral discharge, irritation and dysuria. Urethritis can be broadly classified into gonococcal urethritis (GU) caused by *Neisseria gonorrhoeae* and non-gonococcal urethritis (NGU). Where available, onsite microscopy demonstrates the presence of Gram-negative intracellular diplococci and distinguishes GU from NGU prior to formal laboratory results [1, 2]. NGU incorporates urethritis caused by other organisms including *Chlamydia trachomatis*, *Trichomonas vaginalis*, *Mycoplasma genitalium*, herpes simplex virus (HSV) and adenovirus [1, 2].

The World Health Organization (WHO) lists *N. gonorrhoeae* as one of twelve organisms on the “global priority list of antibiotic-resistant bacteria” [3]. As such, the public health imperative to provide early treatment of GU to disrupt transmission needs to be balanced with the growing threat of antimicrobial resistance. In Australia, rates of gonococcal isolates with decreased susceptibility to ceftriaxone (MIC of > = 0.06) have been recorded as high as 8.8% in 2013, and azithromycin resistance (MIC > = 1.0 mg/L) is also on the rise [4]. Of national concern in Australia is the detection of two cases of multi-drug resistant gonococcal isolates in early 2018 [5].

Syndromic management refers to treatment of clinical syndromes associated with common STIs at time of presentation and was historically developed as a public health strategy in resource-poor areas [6]. A syndromic approach is the mainstay of Australian STI management guidelines for primary care and a similar approach is used in NT and other state-based guidelines [7]. Previous Australian STI guidelines recommended a single dose of oral azithromycin 1 g for syndromic treatment for male urethritis, with an additional dose of intramuscular ceftriaxone 500 mg for treatment of presumed gonorrhoea in high risk groups [8]. The 2018 update to male urethritis guidelines recommend ceftriaxone with doxycycline 100 mg 7 days instead of single dose azithromycin due to macrolide resistance in *Mycoplasma genitalium.* However, ceftriaxone with azithromycin remains the first-line antibiotics for diagnosed *N. gonorrhoeae* genital infections [9].

The Northern Territory (NT), particularly remote NT, has the highest notifications of chlamydia and gonorrhoea in Australia. Centre for Disease Control NT unpublished data from 2018 indicate Darwin urban gonococcal notifications rates to be at 261 per 100,000. In the same year, NT overall gonococcal rates were the highest of all Australian jurisdictions at 859.8 per 100,000 (national average 125.5 per 100,000 in 2018) [10, 11]. Local NT guidelines for urban NT recommend both azithromycin and ceftriaxone for all men presenting with syndromic urethritis [7]. Rural and remote NT gonococcal isolates remain penicillin-sensitive [4] and therefore, local guidelines for rural and remote NT recommend azithromycin and a penicillin-based regime [7].

Larger Australian metropolitan sexual health centres [12–17] and overseas institutions [18–22] have previously published on the aetiology and clinical patterns of male urethritis. However, no equivalent studies are available for the NT.

## Objectives

This study describes the aetiology of male urethritis in urban NT and compares the demographics, risk profiles and clinical features of men with GU to those with NGU. Our primary aim was to identify risk factors for GU in order to better target use of ceftriaxone in empiric treatment of male urethritis. A secondary aim of the study was to compare men with chlamydia to those with other forms of NGU.

## Methods

### Study population

This was a retrospective case-control study using a convenience sample of all consecutive cases of male urethritis presenting to Darwin sexual health clinic from July 2015 to July 2016. A post hoc sample size calculation assuming a prevalence of 25% in controls of a predictor variable (eg. history of urethral discharge), estimated that a total of 105 patients are required in a 1:5 (cases to control) design to detect an odds ratio (OR) difference of at least 4, at 80% power with a two-sided significance level of alpha = 0.05.

Ethics approval was granted by the Menzies School of Health Research (HREC 17–2811). Consent was waivered by the committee as routinely collected and de-identified data was reported.

### Inclusion and exclusion criteria

Potential cases were screened by extracting electronic patient records for 1) all men with a clinical diagnosis of urethritis, and 2) all men treated with azithromycin and ceftriaxone according to clinic protocol for symptomatic urethritis during the study period. Men were included if they had urethral symptoms of dysuria, discomfort or discharge. Asymptomatic men treated as contacts and men with other symptoms related to non-urethral sites were excluded. All representations to clinic within 3 months of initial presentation were considered duplicate cases and were excluded.

Cases were defined as men with GU – that is, symptomatic urethritis and a laboratory diagnosis of *N. gonorrhoeae* on PCR or culture (urine or swab). Controls were symptomatic men with NGU in the same study period.

### Data collection

Electronic records were matched to paper file using the patient’s unique identification code. De-identified data was entered into Excel from patient notes. A standardised clinical template was used for recording patient demographics, history, examination, investigation and management. This included patient-reported risk factors such as number of sexual partners in the past 6 months; history and duration of symptoms such as urethral discharge and dysuria; any abnormalities on genital examination and a description of the abnormal findings; microbiological diagnoses; and antibiotic treatment provided.

### Diagnosis and treatment

Urine samples were collected in all men, and an additional bacterial swab was sent for microscopy, culture and sensitivity for men with urethral discharge on examination. Point of care microscopy and Gram staining was not available in the clinic due to staffing and facility limitations. All samples were tested for *N. gonorrhoeae*, *C. trachomatis*, *T. vaginalis* using Roche cobas® 4800 CT/NG assay. All patients in the study were given ceftriaxone and azithromycin. Where indicated, clinician-dependent additional testing and antibiotic treatment were initiated at the initial consult. For example, some patients presenting with prolonged duration of dysuria received additional testing for *M. genitalium* and were given additional doxycycline treatment as per Australian guidelines for suspected *M. genitalium* [23].

### Statistical analysis

Descriptive statistics and univariate logistic regression explored the correlation between clinical predictors and their association with a diagnosis of GU. Multivariate logistic regression was performed using a backward elimination approach, including marginally significant variables from the univariate analyses (*p* < 0.20) and adjusting for key risk factors highlighted in current published guidelines including age, Aboriginal status, and men with same-sex partners [9, 24]. Variables with non-significant *p*-values were removed manually removed until all remained significantly associated with outcome of interest (*p* < 0.05). Multicollinearity was evaluated prior to variables being included in the final model. Using the same model, a secondary analysis compared those with *C. trachomatis* (CT) to all other patients with NGU.

Unadjusted odds ratios (OR) and adjusted ORs (aOR) were reported with 95% confidence intervals. Where appropriate, Wald chi-square test were used to calculate *P* values and significance was set at *p* = 0.05 throughout. Statistical analysis was performed in Stata (Version 14.0; StataCorp, College Station, Texas).

## Results

### Participants

**Fig. 1:**
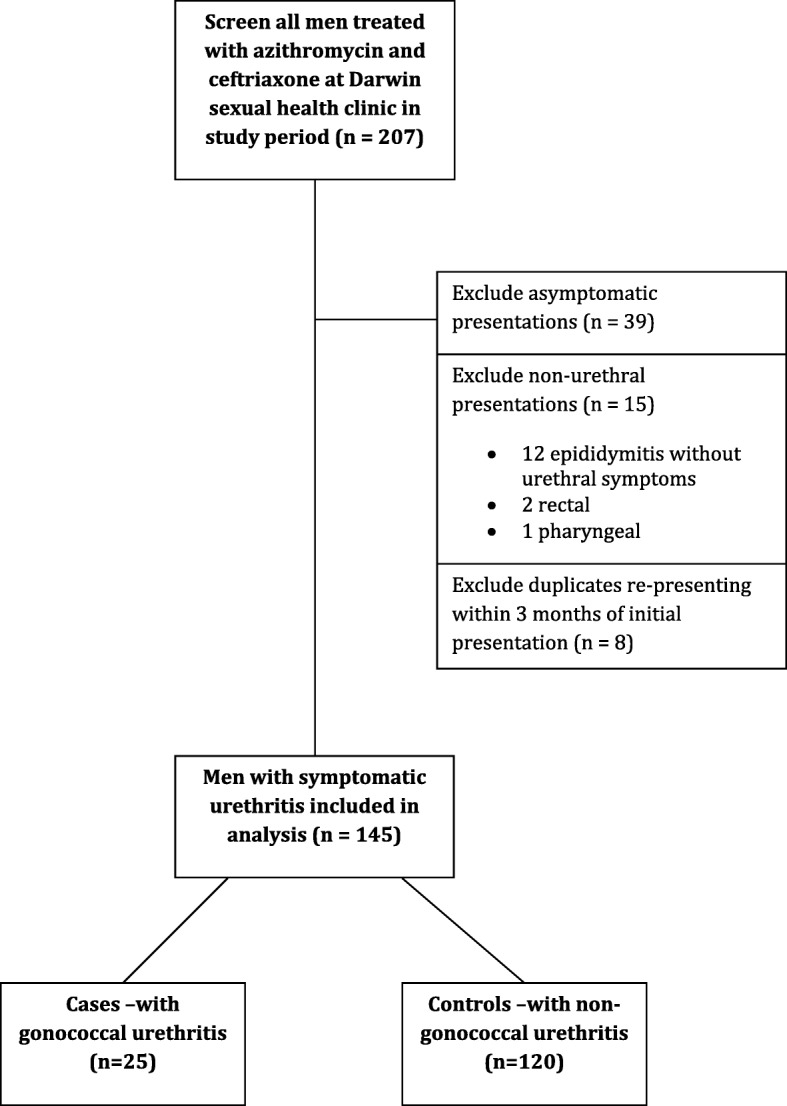
Flow diagram of participants, GU cases and NGU controls

Amongst all men treated with azithromycin and ceftriaxone at the clinic between July 2015 to July 2016, 39 cases were excluded on basis of being treated for asymptomatic infections (eg. as contacts), 15 were excluded due to non-urethral symptomatic infections and 8 duplicate presentations were excluded (Fig. 1). A search for all men with a clinical diagnosis of urethritis revealed no additional cases. In total, *n* = 145 men with symptomatic urethritis were included in the study, with *n* = 25 GU cases and *n* = 120 NGU controls.

### Demographics and risk profile

Patients had a median age of 31 years (IQR, 25 to 40), with 11.3% (*n* = 16) identifying as Aboriginal, and 39.3% (*n* = 57) self reporting as overseas-born. For those born overseas, common countries of birth included UK, Ireland, New Zealand and Germany. Pertinent risk factors and behaviours are reported in Table 1. Being a sexual contact of an individual with a known STI (4.1%, *n* = 6) and having sex with a paid sex work (2.1%, *n* = 3) were uncommon risk factors in the population.

**Table 1:** Demographics, risk factors and clinical features, by diagnosis – GU cases and NGU controls

	Total *n* = 145 (100%)	GU - cases *n* = 25 (17.2%)	NGU - controls *n* = 120 (82.8%)	OR (95% CI)	*aOR (95% CI)*
Demographics					
Median age, years (IQR)	31 (25 to 40)	40 (30 to 50)	30 (24 to 38)		
Age > =30	81/145 (55.9%)	19/25 (76.0%)	62/120 (51.7%)	**3.0** (1.1 to 7.9)^*^	3.0 (0.9 to 9.6)
Aboriginal	16/142 (11.3%)	6/25 (24.0%)	10/117 (8.5%)	**3.4** (1.1 to 10.4)^*^	1.4 (0.3 to 7.0)
Born overseas	57/145 (39.3%)	6/25 (24.0%)	51/120 (42.5%)	0.4 (0.2 to 1.1)	Excluded
Risk factors and behaviours					
Median no. of sexual partners, past 6/12 (IQR)	3 (2 to 6)	3.5 (2 to 7)	3 (2 to 5)		
> =5 sexual partners, past 6/12	55/145 (37.9%)	11/25 (44.0%)	44/120 (36.7%)	1.4 (0.6 to 3.2)	Excluded
Men who have sex with men (MSM)	12/145 (8.3%)	2/25 (8.0%)	10/120 (8.3%)	1.0 (0.2 to 4.7)	0.46 (0.1 to 2.8)
Condom used in most recent encounter	18/145 (12.4%)	1/25 (4.0%)	17/120 (14.2%)	0.3 (0.0 to 2.0)	Excluded
Casual partner in most recent encounter	113/145 (77.9%)	21/25 (84%)	92/120 (75.0%)	1.6 (0.5 to 5.0)	Excluded
Clinical features					
Median duration of symptoms, days (IQR)	5 (2 to 10)	2.5 (1 to 4)	7 (3 to 14)		
> =7 days of symptoms	73/145 (50.3%)	5/25 (20.0%)	68/120 (56.7%)	0.3 (0.1 to 0.9)^*^	Excluded
History of discharge	67/145 (46.2%)	18/25 (72.0%)	49/120 (40.8%)	**3.7** (1.4 to 9.6)^*^	**5.7** (1.4 to 22.6)^*^
History of discharge purulent discharge	39/145 (26.9%)	14/25 (56.0%)	25/120 (20.8%)	**4.8** (2.0 to 11.9)^‡^	Excluded
History of urethral discomfort	135/145 (93.1%)	23/25 (92.0%)	112/120 (93.3%)	0.8 (0.2 to 4.1)	Excluded
Any abnormalities on genital examination	59/116 (50.9%)	19/21 (90.5%)	40/95 (42.1%)	**13.1** (2.9 to 59.3)^‡^	**10.4** (2.1 to 50.8)^*^

Bold font indicate statistically significant result, *p < 0.05, ^‡^*p* < 0.01. Manual backward step method for model building with initial *p* = 0.20 cut-off, adjusting for age > 30, Aboriginal status, and men with same sex partners. Final multivariate model included age > 30, Aboriginal status, men with same sex partners, clinical presentation of discharge and any abnormalities on genital examination

### Clinical presentation

Median duration of symptoms was 5 days (IQR, 2 to 10). Common clinical presentations were dysuria and urethral discharge. Out of the patients who received a physical examination (*n* = 116), 50.9% (*n* = 59) had an abnormal genital examination, with a purulent discharge in 37.3% (*n* = 29) and non-purulent discharge in 28.8% (*n* = 17) of patients. Less common findings included erythema of the glans penis or urethral meatus, testicular tenderness and/or swelling, and abnormal urinalysis.

### Diagnosis and treatment

The two most common organisms identified on nucleic acid amplication test (NAAT) were *C. trachomatis* (23.4%, SE 3.5%) and *N. gonorrhoeae* (17.2%, SE 3.1%). Of men with GU, 16% (*n* = 4) had a B-lactamase resistant strain. Other organisms were identified in 5.5% (SE 1.9%) of patients and these included HSV and *M. genitalium*. No cases of *T. vaginalis* were detected (*n* = 0) and co-infection was uncommon (*n* = 1 had positive NAAT for *N. gonorrhoea* and *C. trachomatis*).

### Primary and secondary outcomes

The main predictors of GU in this study were any abnormalities on genital examination (aOR 10.4, 95% CI 2.1 to 50.8) and a history of urethral discharge (aOR 5.7, 95% CI 1.4 to 22.6). Men over 30 years of age were more likely to have GU in the unadjusted analysis (OR 3.0, 95% CI 1.1 to 7.9) but not in the adjusted model (aOR 3.0, 95% CI 0.9 to 9.6). Aboriginal patients (aOR 3.0, 95% CI 0.9 to 9.6) and those over 30 years of age (aOR 1.4, 95% CI 0.3 to 7.0) were more likely to have GU in the unadjusted analysis, but not in the adjusted model. Men who have sex with men (MSM) were not statistically more likely to have GU in this study. In the secondary analysis, chlamydia was the disease-causing organism in 28.3% (*n* = 34) of those with NGU and modelling did not reveal any profile differences between patients with chlamydia, compared to other patients with NGU (Table 2 in Appendix 1).

## Discussion

Syndromic management has the advantage of providing immediate treatment to interrupt STI transmission, but poses risk for overuse of antibiotics for infections that are not present [25]. WHO recommends countries using syndromic management to conduct local aetiology assessments every several years, in order to inform STI treatment guidelines [26].

The majority of urethritis cases in this study were non-gonococcal and the results support treatment of syndromic urethritis without the addition of ceftriaxone in urban NT. Gonorrhoea accounted for 17.2% of urethritis cases, compared with 30.0% in a previous Japanese study [19] and only 4.2% in an Israeli study [20]. Chlamydia was the most common cause of NGU in this population, identified in 28.3% of symptomatic NGU cases, which was similar to the proportion of *C. trachomatis* (21 to 32%) identified in previous Melbourne-based studies [14, 15] and slightly higher than the proportion (19 to 23%) identified in previous Sydney-based studies [16, 17]. The majority of patients with NGU in our study had no organisms identified (pathogen negative NGU). This is consistent with current understanding of NGU that neither *C. trachomatis* nor *M. genitalium* is detectable in 30–80% of NGU cases [2]. In men with NGU, the role of testing for and treating additional organisms such as *Ureaplasma urealyticum* remains an area of controversy and further research [2, 27].

Australian STI guidelines recommend dual antibiotic therapy with ceftriaxone and azithromycin for suspected GU in high risk populations – including MSM and remote Aboriginal populations [9, 24]. Aboriginal patients were not at significantly higher risk for GU and this may be due to the study being conducted in urban rather than remote NT, where rates of gonorrhoea are extremely high [11]. In our study population, clinical findings of urethral discharge and any abnormalities on genital examination were more prominent predictors of GU than demographic characteristics. Tan et al. similarly reported the importance of clinical findings, describing the use of tissue paper to line underwear in the presence of urethral discharge to be highly specific (99.6%) for a microbiological diagnosis of gonorrhoea [28].

In contrast to GU, men with NGU had neither demographic nor clinical predictors that distinguished those with chlamydia from those with idiopathic urethritis. Previous NGU studies vary widely in patient characteristics associated with pathogen detection [2]. For example, Wetmore et al. reported young age to be a risk factor [18], whereas Rane et al. did not find young age to be a predictor of pathogen detection in NGU [14].

Although this was a retrospective study, consistency for clinical consultation and treatment provided across the study period was high as the clinic used a standardised template to record consultations and an existing treatment protocol for syndromic male urethritis. However, testing and treating for any additional organisms such as *M. genitalium* or HSV was clinician-dependent. Missing data was a limitation for physical examination findings due to genital examinations not being offered or patients declining examination, typically where there was an absence of typical urethritis symptoms. The search method also relied on correct data entry of diagnosis and treatment regimes into the electronic database, which is used as a secondary method of record keeping (paper-based being the primary form) in this clinic.

Ideally microscopy should be available at the time of consultation in addition to history and examination [1, 2, 24]. However, this was not available in Darwin or other rural NT sexual health clinics due to lack of staff and facilities. This issue is not unique to the NT – for example, Libois and De Wit describe that Belgium has a single centre with access to point of care microscopy, highlighting the need for existing US and European guidelines to provide clearer direction on ceftriaxone use for syndromic urethritis therapy for the many settings where microscopy is not available [29]. Development of combined point of care assays for *C. trachomatis* and *N. gonorrhoeae* NAAT offer promising alternatives where laboratory equipment or human resourcing is limited; future use in Australia would need to take into implications for workflow and changes to existing treatment algorithms [25, 30].

This study demonstrates that despite the overall extremely high rates of gonococcus in NT [11], urban NT should adopt national guidelines where ceftriaxone is only used in high-risk cases for gonorrhoea [9, 24]. Although this is a single site study in Darwin, the recommendation is generalisable to other primary care settings in urban NT where rates of gonorrhoea in general practice can be expected to be lower than that of the specialised Darwin sexual heath clinic. Rural and remote NT populations remain high risk and should continue to receive empiric treatment for gonorrhoea using a penicillin-based regime [7, 11].

## Conclusion

We recommend revising the current NT guidelines to be consistent with national guidelines to use ceftriaxone for empiric management of syndromic urethritis only in high-risk patients. In this urban NT setting, high risk was associated more closely with clinical features than traditional demographic factors, emphasising the importance of offering a genital examination to risk stratify patients. Although Australian STI guidelines continue to rely heavily on initial syndromic management, the development of point of care NAAT presents future options for improved antimicrobial stewardship.

## Data Availability

The datasets used and/or analysed during the current study are available from the corresponding author on reasonable request.

## Appendix

**Table 2:** Demographics, risk factors and clinical features, by diagnosis – CT cases and other NGU (controls)

	CT *n* = 34 (28.3%)	Other NGU *n* = 86 (71.7%)	OR (95% CI)	aOR (95% CI)
Demographics				
Median age, years (IQR)	28.5 (23 to 34)	31 (25 to 38)		
Age > =30	15/34 (76.0%)	47/86 (54.7%)	0.7 (0.3 to 1.4)	0.6 (0.2 to 1.6)
Aboriginal	2/33 (6.1%)	8/84 (9.5%)	0.6 (0.1 to 3.1)	0.9 (0.2 to 5.1)
Born overseas	12/34 (35.3%)	39/86 (45.4%)	0.7 (0.3 to 1.5)	Excluded
Risk factors and behaviours				
Median no. of sexual partners, past 6/12 (IQR)	3 (2 to 4)	3.5 (2 to 5.5)		Excluded
> =5 sexual partners, past 6/12	9/34 (26.5%)	35/86 (40.7)	0.5 (0.2 to 1.3)	
Sex with same-sex partner	0/34 (0%)	10/86 (11.6%)	^a^	^a^
Condom used in most recent encounter	6/34 (17.7%)	11/86 (12.8%)	1.5 (0.4 to 4.3)	Excluded
Casual partner in most recent encounter	27/34 (79.4%)	65/86 (75.6%)	1.2 (0.5 to 3.3)	Excluded
Clinical features				
Median duration of symptoms, days (IQR)	7 (5 to 14)	5 (2 to 12)		Excluded
> =7 days of symptoms	26/34 (76.5%)	42/86 (48.8%)	**3.4**(1.3 to 8.4)	
History of discharge	12/34 (35.3%)	37/86 (43.0%)	0.7 (0.3 to 1.6)	0.8 (0.3 to 2.1)
History of discharge purulent discharge	4/34 (11.8%)	21/86 (24.4%)	0.4 (0.1 to 1.3)	Excluded
History of urethral discomfort	31/34 (91.2%)	81/86 (94.2%)	0.6 (0.1 to 2.8)	Excluded
Any abnormalities on genital examination	10/25 (40.0%)	30/70 (42.9%)	0.9 (0.4 to 2.3)	1.01 (0.4 to 2.7)

Bold font indicate statistically significant result, p<0.05^a^Excluded due to cell size < 1. Final multivariate model included age > 30, Aboriginal status, men with same sex partners, clinical presentation of discharge and any abnormalities on genital examination

## Abbreviations

CT: *Chlamydia trachomatis*
GU: Gonococcal urethritis
HSV: Herpes simplex virus
MSM: Men who have sex with men
NAAT: Nucleic acid amplification test
NGU: Non-gonococcal urethritis
NT: Northern Territory
WHO): World Health Organization
